# Hemocytes: A Useful Tool for Assessing the Toxicity of Microplastics, Heavy Metals, and Pesticides on Aquatic Invertebrates

**DOI:** 10.3390/ijerph192416830

**Published:** 2022-12-15

**Authors:** Federica Impellitteri, Alexandrina-Stefania Curpăn, Gabriel Plăvan, Alin Ciobica, Caterina Faggio

**Affiliations:** 1 Department of Veterinary Sciences, Polo Universitario dell’Annunziata, University of Messina, 98168 Messina, Italy; 2 Department of Biology, Faculty of Biology, “Alexandru Ioan Cuza” University of Iasi, Carol I Avenue, 20A, 700505 Iasi, Romania; 3 Department of Research, Faculty of Biology, “Alexandru Ioan Cuza” University of Iasi, Carol I Avenue, 20A, 700506 Iasi, Romania; 4 Department of Chemical, Biological, Pharmaceutical, and Environmental Sciences, University of Messina, Viale Ferdinando Stagno D’Alcontres 31, 98166 Messina, Italy

**Keywords:** hemolymph, phagocytosis, model organisms, xenobiotic, pollution, invertebrates

## Abstract

Invertebrates have long been an important tool for assessing water pollution due to their characteristics as intermediate consumers in aquatic ecosystem food chains. Most of the time, the effects of contaminants are measured by their effect on oxidative status or by mortality, although there already exists an easier tool—hemocytes. Hemocytes are circulating cells with a very important role in the immune system of invertebrates, which can be found within the hemolymph, analogous to the blood in vertebrates. The collection of hemolymph samples is easy, fast, minimally invasive, and poses no danger to the life of invertebrates. The purpose of this review was to highlight the advantages of using hemolymph for toxicity assays of various substances, including heavy metals, micro- and nano-plastics, pesticides, hydrocarbons, and oil spills. A literature search was conducted for this purpose using the most common and most often used databases, with a focus on the most recent and relevant studies. Bivalve mollusks, crustaceans, and gastropods were chosen for this investigation. This review found a growing number of studies choosing to use hemolymph as the standard methodology for toxicology assays, confirming their qualities as reliable tools.

## 1. Introduction

### 1.1. Hemolymph

All multicellular organisms have various defense systems against invading micro-organisms and against everything that the cell recognizes as “non-self”. These defense systems are essential for their survival and maintenance. An important role here has the hemocytes—cells capable of phagocytosis—that are found in a liquid called hemolymph. This review aims to focus attention and highlight the advantages of using this liquid and its constituent cells on invertebrates. Indeed, while scientific studies using vertebrates as experimental models have been a cornerstone of scientific progress in recent centuries, ethical concerns about their use have led researchers to seek alternatives [1,2]. Furthermore, invertebrates can replace vertebrate counterparts in many areas of research, including toxicological studies, bridging the gap between in vitro models and vertebrate animal studies [3]. Due to their close resemblance to vertebrate responses, rapid reproduction rate, and low cost, invertebrates have become important organisms for toxicological screening. In addition, they have a shorter lifespan and smaller size, making them more manageable and easier to enclose, while remaining interesting and novel compared to more traditional animals such as mammals and birds [1,3].

Bivalves are among the most used invertebrates in the research context. For bivalves, the term hemolymph is used to describe a colorless fluid composed of blood cells, called hemocytes, and plasma, the hemolymph without cells [4]. The blood volume of bivalves is quite large, and its cellular characterization can be performed using a 1 mL sample of hemolymph [5]. In addition to hemocytes, the hemolymph is composed of water, inorganic (calcium, magnesium, and sodium) and organic compounds (carbohydrates, proteins, lipids, and enzymes); therefore, it is responsible for transporting substances essential to the life of the mussel, including oxygen [6].

Bivalve’s hemolymph is widely used in several research fields. The hemolymph of the Mediterranean mussel, *Mytilus galloprovincialis*, for example, is used as an indicator of bioaccumulation in environmental studies concerning heavy metals such as zinc and cadmium [7,8] or even in toxicity assays of neonicotinoid insecticides [9]. Mussels are sessile filter feeders, i.e., they can continuously filter the surrounding water. This characteristic makes them an excellent model organism for ecotoxicological and eco-physiological studies; for example, most of the studies on absorption, on the biological consequences of ingested microplastics, and many of the toxicity tests of heavy metals can all be carried out thanks to the collection of hemolymph. As far as the collection is concerned, this can vary depending on the nature of the research: hemolymph can be extracted by minimally invasive (non-lethal) procedures, where the organism survives after collection. The main site of hemolymph collection in bivalves is generally the adductor muscle [8,9] (anterior or posterior, depending on the species), although less common, it also can be extracted from the ventricle of the heart [4]. The shell of bivalves, such as *Elliptio complanata* (a freshwater bivalve mollusk), is frequently kept open with forceps to allow hemolymph collection. The adductor muscle is plainly visible at this phase, seen in a highly reflecting shimmering white surface between the opening shells. In a 2005 investigation, a syringe fitted with a 5/8-inch 25 ga needle was then employed, injecting it along a line parallel to the front edge of the shell valves. A successful extraction from a 50 g organism gives around 0.5 mL of fluid in 30 s [10]. In this very interesting research, the chemical composition of the hemolymph was also evaluated, highlighting the differences that arose in relation to the collection site. It was ascertained (Table 1) that hemolymph varied minimally according to the collection site, which is why the adductor muscle appears to be a better alternative than the more difficult cardiac chamber for hemolymph collection. Only calcium and cell number indicated statistically significant variations between fluid types, with adductor muscle fluid concentrations being higher than equivalent cardiac fluids [10].

In addition to bivalve mollusks, the hemolymph of some crustaceans and gastropods have now been characterized for a few years, thus, expanding the available model organisms and making toxicological assays easier. Crustacean hemolymph coats and surrounds all cells in the animal’s interior (hemocoel). Hemocyanin, a copper-based protein that turns blue when oxygenated, replaces the iron-based hemoglobin found in vertebrate red blood cells, giving hemolymph a blue-green color rather than the red appearance of vertebrate blood [11]. Collecting hemolymph from crustaceans such as *Carcinus aestuarii* is a rapid and simple procedure. Crabs are anesthetized on ice for 10 min, and hemolymph is collected using a syringe from the unsclerotized membrane of the walking legs and placed in Eppendorf tubes [12,13]. Hemolymph from gastropods such as *Haliotis tuberculata* can be taken from the cephalic arterial sinus located in the anterior part of the muscle and again using a syringe [14].

### 1.2. Hemocytes

Hemocytes are the cells responsible for defending the organism from everything foreign and harmful to the animal. They are the first line of defense against pathogens, recognizing and eliminating them through phagocytosis and the secretion of antimicrobial peptides, various humoral factors, and reactive oxygen intermediates [15].

For *Mytilus galloprovincialis*, two types of hemocyte cells have been identified: hyalinocytes and granulocytes [15]. Hyalinocytes are smaller than granulocytes, have large nuclei and little cytoplasm, and are commonly known to be granule-free. Granulocytes, on the other hand, are larger with a smaller nucleus and numerous cytoplasmic granules. Three classes of granulocytes have been distinguished based on the staining properties of their cytoplasmic granules. There are acidophilic granulocytes, basophilic granulocytes, and cells containing both acidic and basic granules. Granulocytes are also capable of producing pseudopodia and possess phagocytic activity [15,16].

**Figure 1:**
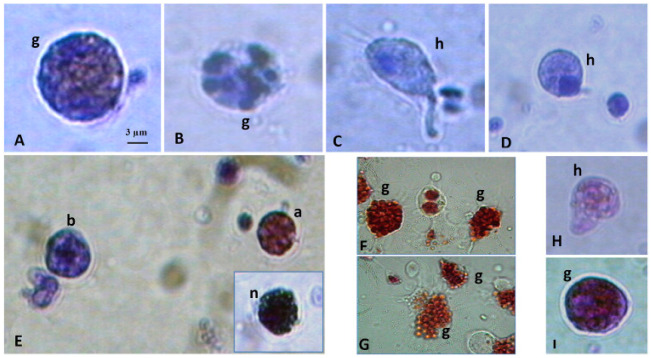
*Pinna nobilis* (bivalve) hemocytes under a light microscope (100×). (**A**–**D**): Giemsa staining; (**E**): Pappenheim’s panoptical and Ehrlich’s triacid mixture (inset) staining; (**F**,**G**): neutral red staining; and (**H**,**I**): PAS reaction. g: granulocyte; h: hyalinocyte; b: basophil; a: acidophil; and n: neutrophil [16].

**Figure 2:**
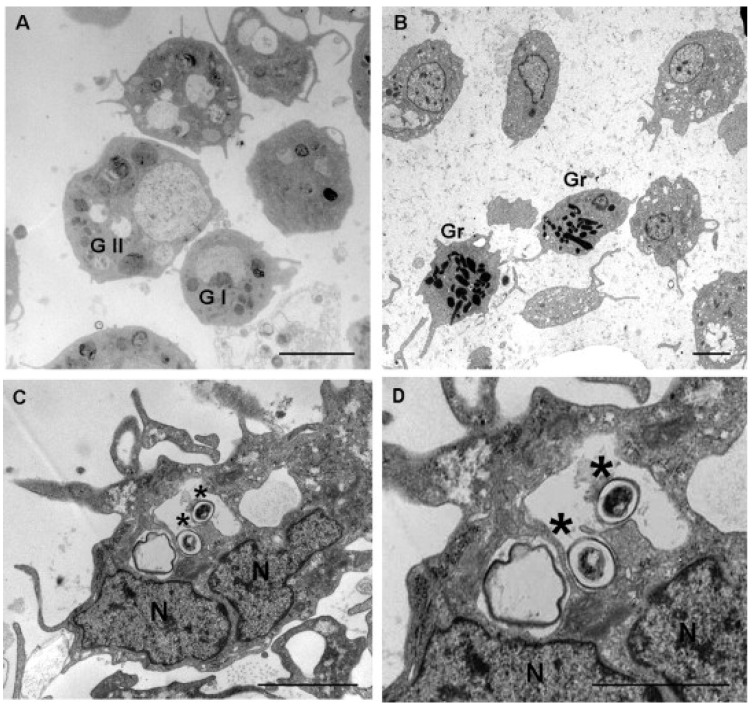
Hemocytes of *P. canaliculata* (freshwater snail) observed at TEM. (**A**) Group I (G I) and II (G II) agranular cells, both with regular shapes but different dimensions, are observable. (**B**) Group II (Gr) granular hemocytes show electron-dense granules. (**C**,**D**) Phagocytosis of bacteria (*) is observed in group II cells. N—nucleus [17].

Moreover, according to a 2016 study [16], all the cell types of the hemolymph found in the bivalve *Pinna nobilis* (Figure 1) were able to produce superoxide anions and hydrolase as a spontaneous defensive response to the presence of non-self-material. They can also conduct the phagocytosis of a non-self-substance, as shown in a study conducted on the freshwater snail *Pomacea caniculata* in 2013 [17] (Figure 2). In this process, there are most likely two types of receptors involved: those that bind the integral surface components of micro-organisms without any humoral factors and those that identify serum components of the hemolymph that coat foreign particles and act as opsonins [16]. The research team focused on two types of cells in their investigation of P*. caniculata*. Small, spherical cells with a big nucleus and smooth, agranular, basophilic cytoplasm make up group I. They are rather steady in terms of stainability and form, but their percentage decreases following hemolymph collection, most likely due to their increasing size. Heat-inactivated *Escherichia coli* does not appear to be phagocytized by group I cells (used in this study as an external organism to test the phagocytic abilities of hemocytes). Group II cells are heterogeneous in that they are bigger in size, less uniform in shape, and have a lower nucleus/cytoplasm ratio than group I cells. The group II cells may also bind to and phagocytize micro-organisms [17].

**Figure 3:**
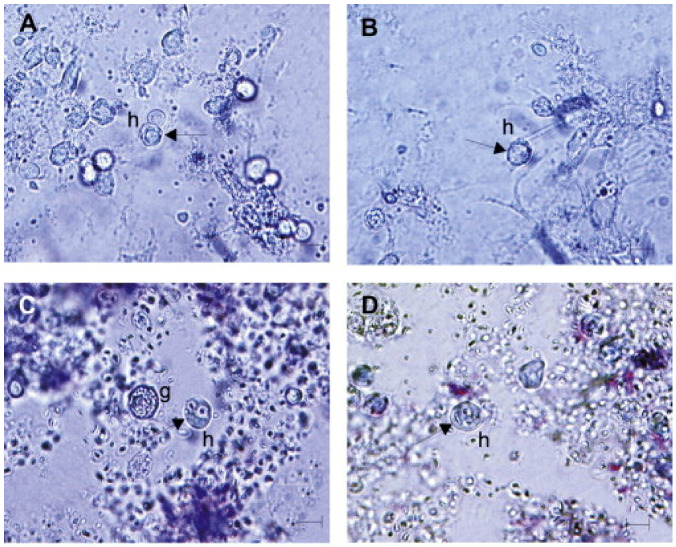
Hyalinocytes endowed with phagocytic capacity, and incorporate yeast cells (arrow) (**A**,**B**) or Zymosan cells (**C**,**D**) in a study conducted on *Carcinus aestuarii* (Crustacea, Decapoda) [13]; g—granulocytes, h—hyalocytes.

Regarding crustacean defense systems, they are fully dependent on the innate immune system, which is activated when pathogen-associated molecular patterns are identified by soluble proteins or the host cell surface, triggering cellular or humoral effector mechanisms to eliminate invading pathogens [12]. Again, hemocytes play an important role in this defensive system. They are similar to vertebrate leukocytes in that they are primarily involved in the detection and removal of non-self-materials [13]. Although one of the most disputed issues is the morphological classification of crustacean hemocytes, most species recognize three types of circulating hemocytes: hyalinocytes, semigranulocytes with small granules, and granulocytes with numerous cytoplasmic granules. Hyalinocytes are responsible for phagocytosis (Figure 3), semigranulocytes for minute particle encapsulation, and granulocytes for the pro-phenoloxidase (proPO) system, which is an important component of cellular defense systems.

**Figure 4:**
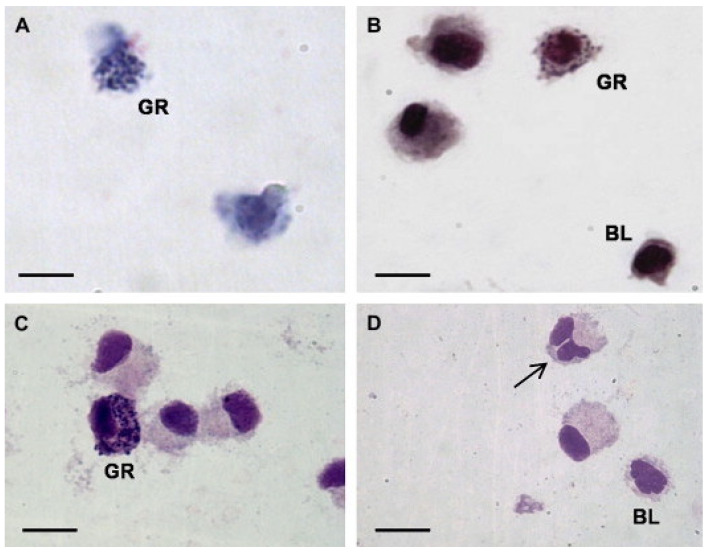
Light micrographs of hemocytes of the gastropod *H. tuberculata* (**A**). Hemocytes stained with Giemsa (**B**). Hemocytes stained with MGG (**C**,**D**) [14]. GR, granulocytes; BL, blast-like cell. All other cells are large hyalinocytes. Take notice of the bilobulated nucleus (arrow).

A 2008 study on the gastropod *Haliotis tuberculata* revealed only granulocytes and hyalinocytes by using Giemsa, May–Grünwald Giemsa, or Hemacolor^®^ staining (Figure 4). Granulocytes, which made up less than 1% of the centrifuged cells, were round or ovoid in form, with centered, round, or oval nuclei, and were frequently lobulated. Many blue basophilic granules were seen in the cytoplasm. Hyalinocytes, which made up the vast portion of the hemocyte population (more than 99% in cytocentrifuged cells), were classified as small or big based on their size. Large cells showed many cytoplasmic vacuoles that did not stain, whereas tiny cells had very little cytoplasm [14].

### 1.3. Model Organisms

The term “model organism” has become ubiquitous in biological studies and is increasingly used to describe any experimental organism that is useful for studying a range of systems and processes that occur in living organisms, including genetics, development, physiology, evolution, and ecology. Model organisms must have specific genetic characteristics. They must be small in physical and genomic size, with low breeding, maintenance, and transport costs, a short reproduction time and life cycles, and must, finally, possess high fertility rates [18].

Among those model organisms useful in ecotoxicology studies, invertebrates occupy an essential role as intermediate consumers in the food chains of aquatic ecosystems. For this reason, aquatic organisms can be excellent bioindicators of marine and freshwater quality [19,20,21]. Moreover, using these organisms can also be useful in determining the toxicological risk faced by other marine organisms, and even humans, who may come into contact with pollutants through the ingestion of contaminated edible species, such as the bivalve *Mytilus edulis* [4].

Bivalves are excellent model organisms as they present the above-mentioned characteristics, and the hemolymph can be easily collected with a syringe from the anterior adductor muscle after opening the valves of the specimen [22].

It is interesting to investigate the immune system of these organisms because it appears useful for assessing the survival rate of both individuals and populations and as a basis for a better understanding of the processes that have been preserved during evolution, from innate to acquired immunity, typical of vertebrates [23].

*Mytilus galloprovincialis* is among the most studied mollusk, used as a biological indicator because of its great capacity to absorb and bioaccumulate many toxic substances. When an organism is exposed to xenobiotics, its immune response can influence other levels of the biological spectrum, such as growth rate, food uptake, and reproduction through changes in histopathology and bioenergetics. In addition, mussels such as *M. galloprovincialis* are quite tolerant to environmental changes, appearing less affected or undamaged by syndromes and infectious agents that afflict other bivalves. This resistance is probably because of a robust immune system that kills pathogens [9].

## 2. Hemocytes’ Response to Xenobiotics

Xenobiotics are compounds with toxic effects resulting from waste or the inappropriate disposal of products. These substances reach aquatic ecosystems through various routes, directly through the discharge of chemical compounds into water or more indirectly through residues that have accumulated into the soil, and as a consequence of a flood, rains, or land runoff, they reach water sources. Organisms affected by these xenobiotics exhibit increased DNA damage, increased reactive oxygen species’ production, and disturbances in the hemolymph composition and the number of existing hemocytes [24,25].

The present paper is focused on the effects of different classes of xenobiotics on the hemocytes and hemolymph of several aquatic invertebrates. A literature search was carried out throughout the year 2022 in the most common and often used databases (MEDLINE, HINDAWI, and Google Scholar) and included articles published after the year 2000 with a focus on the most recent, relevant ones. The articles were found by using the keywords (xenobiotics, phagocytosis, hemocyte, hemolymph, microplastics, heavy metals, hydrocarbons, and pesticides) under different combinations and specificity.

Subsequently, the articles were selected by the keywords in the title or abstract and their connection to invertebrates and cell viability, lysosomal membrane stability, and hemocytes’ biochemical parameters. Articles using species already established as aquatic animal models (e.g., *Danio rerio*) and articles that did not measure the total hemocyte count or the phagocytic activity were excluded. However, articles measuring only the total hemocyte count (THC), phagocytosis, or lysosomal membrane stability (LMS) were included. For the present article, a total of 39 references were selected: 17 referring to heavy metals, 12 to pesticides, 4 to hydrocarbons, and 7 to plastics. The results are summarized in Table 2.

### 2.1. Heavy Metals

Sewage discharges, farming, electronic waste, mining activities, and even natural climate changes are some of the main sources of heavy metal contamination in the aquatic environment [62,63]. Two of the properties that make heavy metals a serious threat to the environment are their ease of dissolving into water and subsequently being absorbed by the living organisms inhabiting the medium and their lack of biodegradability [64]. At low concentrations, most heavy metals are essential to the life of invertebrates, helping with bone or shell formation or muscle development, but when they reach high concentrations, they can lead to severe physiological changes and even the death of the organism. The bioaccumulation of these substances not only represents a danger for the invertebrates but also becomes a threat to human health upon their consumption since most of the changes they trigger are not obvious to the untrained eye [58,59].

One interesting study investigating the effects of organic mercury on the hemolymph of *M. galloprovincialis* reported that a concentration of 10^−5^ M could induce morphological changes by changing the hemocyte’s shape and number and also reducing its ability to conduct phagocytosis [25]. In an older study about ionic regulation in the crayfish *Orconectes propinquus* upon mercury exposure for a 14-day [27] period, the author observed a significant decrease in Na^+^ and Ca^+^, with the difference that the Ca^+^ concentration increased after the depuration period [27]. In a recent study [18] that examined the hemolymph of the crustacean *Portunus pelagicus*, it was found that exposure to cadmium chloride and mercuric chloride increased the concentration of glucose in the blood. The 48 h and 24 h exposures showed a higher glucose concentration. One interesting in vitro study noted the different effects of mercury on the phagocytosis of *M. edulis*, and at concentrations ranging from 10^−6^ to 10^−3^ M, phagocytosis significantly decreased, while concentrations of 10^−9^ M to 10^−7^ M determined an increase [29].

Copper has long been known to negatively impact the health status of invertebrates, as one study on its effects at concentrations of 10, 60, and 110 µg/L on the bivalve *Tapes philippinarum* presented. Copper was capable of significantly reducing phagocytosis and lysosomal membrane stability [30]. Whereas another study significantly altered 25 metabolites of the bivalve hemolymph of *Perna canaliculus* exposed to a high concentration of Cu^2+^ (125.0 µM) [31]. Huang et al. [32] and his team conducted a multiapproach study to analyze the effect of copper exposure (0, 10, and 50 μg/L) at two different pH levels (7.7 and 8.1), and they noted that copper significantly decreased the total hemocyte count at both pH levels; however, copper, together with low pH, led to a significantly higher phagocytic activity in the bivalve *Crassostrea rivularis* hemocyte.

Cadmium possesses immunotoxic effects on invertebrates, especially on mussels, which are filter feeders and have an open circulatory system [65]. Concentrations of 10^−6^ to 10^−3^ of cadmium significantly reduced hemocyte viability and phagocytosis activity in a dose-dependent manner in the hemocytes of the mussel *Dreissena polymorpha* [33]. Whereas in the bivalve *Crassostrea virginica*, a concentration of 50 µmol L^−1^ significantly increased apoptosis [32]. Similar results were observed in *T. philippinarum* [24]. For *Mytilus galloprovincialis*, cadmium exposure significantly decreased lysosomal membrane stability at a concentration of 10 µg/L [34], while in *M. edulis* [35], a concentration of 10^−3^ M for 21 h significantly decreased both phagocytosis and hemocyte viability. For the crustacean *Sinopotamon henanense* [38], similar results were obtained at concentrations of 58 and 116 mg L^−1^ alongside decreased protein content. However, one study on the decapod crustacean *Litopenaeu vannamei* showed that the effect of cadmium at all concentrations was a short-time response, which decreased over time in terms of protein content [36].

Lead is another heavy metal that is an environmental contaminant as a result of anthropogenic activities or natural events. A short time exposure (96 h) to different concentrations (10 to 500 mg L^−1^) of lead led to the maximum decrease of total hemocyte count between 5 and 8 h of exposure in the crustacean *Palaemon elegans* [37]. Whereas for the bivalve *Lamellidens marginalis*, a concentration of 50 mg/L of lead nitrate determined a decreased mean phagocytic index and increased mean mortality index in a preliminary study [38]. Mosher et al. [39] conducted an interesting study on the effects of lead on hemolymph ion concentrations for the bivalve *Elliptio complanata*, and they illustrated that exposure to 245 µg/L of lead determined significantly elevated Ca^2+^ levels, decreased Na^+^ levels, but had no effect on Cl^−^ and K^+^.

### 2.2. Pesticides

With the increased demand for vegetables, grains, and fruits due to the continuously expanding world population, the use of pesticides to protect crops against diseases or predators has increased as well [66]. Often these substances reach freshwater ecosystems, affecting the organisms inhabiting them. Pesticides can be naturally derived or synthetic and can have a specific or broad-spectrum action. The main classes of pesticides are insecticides, herbicides, and fungicides [67]. Pesticides or insecticides that flow into rivers can affect an organisms’ morphology, function, and health [68]. The issue is even more pressing since, nowadays, an aquatic ecosystem is contaminated by a combination of pesticides, and organisms such as mussels, for example, can bioaccumulate more than one substance [63].

The toxic effects of pyrethroids (insecticides) are well studied, especially for mammals, with more and more increasing evidence of their effect on invertebrates surfacing. One study that analyzed the effects of cypermethrin (a neurotoxic insecticide used for lettuce and cotton) for the gastropod *Bellamya bengalensis* and fenvalerate (a broad-spectrum insecticide) for *L. marginalis* noted an increased total hemocyte count in both cases at all concentrations used [40]. Interestingly, one team investigated the effect of a mixture of 14 pesticides (carbaryl, fosetyl, alachlor, metolachlor, atrazine, terbuthylazine, diuron, AMPA, bentazon, tebuconazole, imidacloprid, mancozeb, and metaldehyde) with a final concentration of 0.5% on the hemocytes of the bivalve *Crassostrea gigas* [50]. The results suggested there was no difference in hemocyte activities or mortality; however, a difference based on the incubation period was identified. Pyrazosulfuron-ethyl has been deemed non-toxic (at concentrations from 0 to 1000 µg L^−1^) to juveniles of *Litopenaeus vannamei*; however, it produced significant differences in the total hemocyte count compared to the control, while permethrin was highly lethal for juveniles at concentrations ranging from 0 to 10 µg L^−1^ (both pesticides are used for rice cultures) [41].

Concentrations of 10, 100, and 1000 µg of glyphosate (a broad-spectrum herbicide) can significantly decrease the total number of hemocytes upon exposure for 7 days and significantly increase their volume at two of the concentrations tested (100 and 1000 µg/L) in *Ruditapes philippinarum* [42]. Another study on the gastropod *Haliotis tuberculata* showed no effect on phagocytic activity of lysosomal membrane stability even after exposure to the highest doses (10,000 and 100,000 µg L^−1^) [43]. A research group established that a dose of 400 mg/L of glyphosate is lethal for the crustacean *Eriocheir sinensis*, and they also noted a significant decrease in THC after exposure to different glyphosate concentrations, and the count continued to decline after 24 h exposure to concentrations of 44 and 98 mg/L. Glyphosate also significantly decreased phagocytosis [44].

Thiocarbamates are fungicides and one of the most common types of contaminations known to have neurotoxic, genotoxic, pro-oxidative, and endocrine disruption activities. One study using the bivalve mussels *Anodonta anatina* and a concentration of 91 µg L^−1^ of thiocarbamates (mancozeb and propamocarb) observed a significantly decreased lysosomal membrane stability between control groups and propamocarb [45,46].

Chlorpyrifos is a pesticide in the organophosphate class and is commonly used against soil-borne insect pests. Its toxic effects have not been yet fully appreciated in different types of model organisms. For this reason, Chinonso et al. [47] conducted a study on the crustacean *Cardiosoma armatum* and observed a decrease in total hemocyte count at concentrations of 0.006, 0.03, and 0.06 mg/L. Concentrations of 1 and 7.5 µg L^−1^ had no significant effect on the hemocyte number or lysosomal membrane stability for the gastropods *Biomphalaria glabrata* and *Planorbarius corneus*, but it did increase phagocytosis for all exposed groups [48]. For the crustacean *Parathelphusa jacquemontii*, exposure to increasing concentrations of chlorpyrifos and cypermethrin (0.0187 and 0.0374 ppm) drastically reduced the total number of circulating hemocytes and reduced phagocytosis at 7 and 21 days [49].

Thiacloprid is a neuroactive insecticide from the class of neonicotinoids—pesticides similar in structure to nicotine. Neonicotinoids are competitors of acetylcholine, and their action mechanism is by signal transmission disruption. A very interesting study on the effects of thiacloprid on the hemolymph and digestive cells of *M. galloprovincialis* was conducted, on top of analyzing the lysosomal membrane stability and the total hemocyte count; they also investigated the changes in terms of hemolymph biochemical parameters. They noted the high stability of the membrane at concentrations of 4.5 and 450 µg L^−1^; also, both concentrations led to a significant increase in Ca^2+^ and NH_3_ and a decrease in PHOS levels after 10 days of exposure. Moreover, a concentration of 450 µg L^−1^ also determined an increase in Mg^2+^ and a decrease in urea content [2]. A previous study of the same team obtained similar results at different concentrations (1, 5, and 10 mg L^−1^) with a significant decrease in Cl^−^ and K^+^ and a significant increase in glucose [5].

### 2.3. Hydrocarbons and Oil Spills

Hydrocarbons are one of the most studied contaminants of the aquatic environment. Hydrocarbons, especially polycyclic aromatic hydrocarbons are often the result of processes such as oil spills and combustion processes. High concentrations of hydrocarbons, such as 10^−3^ and 10^−5^ mg mL^−1^, are often found in aquatic environments after oil spills, and their toxicity usually comes from the intermediates formed during the degradation processes [50,54,69]. A study using these concentrations, and also 10^−7^ and 10^−9^ mg mL^−1^, conducted on *C. gigas* in vitro reported decreased phagocytosis upon 24 h incubation with fluorene, pyrene, and pure heavy fuel oil [50]. A study conducted a year after the Hebei Spirit oil spill in Korea, on the same species observed decreased total hemocyte count and phagocytic activity [51]. Interestingly, in the bivalve *Chlamys farreri*, lower concentrations of benzo(a)pyrene—0.5, 1, 2.5, and 7.5 µg L^−1^—were enough to significantly decrease total hemocyte count, phagocytic activity, and even antibacterial activity [52]. For the gastropod *Haliotis diversicolor*, concentrations of 0.01, 0.02, 0.04, and 0.08 mg L^−1^ of benzo(a)pyrene significantly decreased the total number of circulating hemocytes and phagocytic activity at higher concentrations [53]. Phenanthrene and anthracene produced significant effects on lysosomal toxicity alone and in combination at 100 µM on *M. galloprovincialis* [70].

### 2.4. Micro- and Nano-Plastics

One of the major pollution sources and a major cause of concern in modern society is contamination due to plastics. Plastics are used in a plethora of industries, including the construction, food, and car industry and even the pharmaceutical industry [54,55]. The major issue regarding plastic pollution is their very long time of degradation with very limited options for natural mechanisms. Plastic degradations lead to the formation of microplastics followed by the degradation of even smaller particles, nano-plastics [56]. Micro- and nano-plastics can easily contaminate soil and water; vertebrates and invertebrates can bio-accumulate them, leading to different pathological manifestations [57].

In a comparative study on the effect of polystyrene micro and nano-plastics using hemocytes from *M. galloprovincialis*, the authors observed a significant decrease in lysosomal membrane stability and phagocytic activity at 1.5, 15, and 150 ng/L microplastic concentration exposure [54]. *Mytilus edulis* and *Mytilus galloprovincialis* exposed to microplastic beads, 2 and 6 µm in size, exhibited reduced total hemocyte count and significantly higher phagocytosis capacity compared to the control group [55]. Whereas an older study conducted on *M. edulis* investigating the effects of ingestion of polystyrene microplastics found no significant differences in terms of phagocytosis or the viability of the hemolymph; however, the authors noted some significant differences between the collection times of samples (day 6 and 12 for viability, day 12 and 48 for phagocytic activity) [56]. For *Crassostrea gigas* exposed for 2 months to micro-polystyrene spheres of 2, 6 µm, 0.023 mgL^−1^ and used for assessing its effect on its reproductive capabilities, microplastics affected the size of hyalinocytes and granulocytes in exposed oysters, significantly increasing their size [62]. Another comparative study on the effects of the exposure to 500 ngmL^−1^ of 20 µm polystyrene microplastics or 50 nm nano-plastics for 24 h or 7 days in *M. edulis* observed no effects in terms of lysosomal membrane stability and immune response upon exposure to microplastics, whereas nano-plastics produced significant differences [57].

When it comes to nano-plastics, a study evaluating the effects of polystyrene nano-plastics on *M. galloprovincialis* showed a significant decrease in phagocytic activity at 10 mgL^−1^ of 1 µm polystyrene nano-plastics at the first time of sampling (3 h) and increased hemocyte apoptosis [58]; whereas another study observed no significant differences in the number of circulating hemocytes or the hemocyte subpopulations and phagocytic activity upon exposure to 10 µg/L nano-plastics for 24 h but significantly decreased lysosomal membrane stability [59]. A decreased lysosomal membrane stability and phagocytic activity were also observed upon exposure to different concentrations of polystyrene nano-plastics (1, 5, and 50 µg/mL) in *M. galloprovincialis* [60].

## 3. Conclusions

Environmental pollution becomes more and more of a pressing matter for today’s society with causes rooted in the increasing global population and food demand or inappropriate disposal of waste. Hemocytes and hemolymph are complex components of the invertebrate’s physiology that can be successfully used as biomarkers for the devastating effects of different contaminants. Collection of hemolymphs is easy, minimally invasive, and poses no danger to the life of invertebrates, providing a plethora of information regarding their health status, the oxidative and immune response when facing the contaminant, the reproductive abilities, and the toxic manifestations of different substances. The use of hemocyte analysis has the potential to become a standard methodology for toxicology assays since it is simple, fast, and cost-effective. Especially, as highlighted by this review, the studies that most make use of hemolymph as a tool in toxicology assays are those investigating the effects of heavy metals and pesticides. To date, on the other hand, research using this very useful tool on microplastics and nano plastics, as well as toxicological studies on hydrocarbons and oil spills, are very few and could be expanded considering the great benefits of it. Although hemocyte-centered studies on the alterations produced by different xenobiotics are relatively small, it was still possible to observe an increasing trend in the number of publications based on pollutant class. This supports the idea that hemocyte and hemolymph analysis is a reliable tool and allows the use of multiple aquatic organisms to obtain accurate information.

## Figures and Tables

**Table 1:** Results of Friedman’s test of differences between harvest sites in a 2005 study of *Elliptio complanata* [10]. Median values for adductor muscle and ventricular fluids are presented, as well as Friedman’s S statistic and related *p*-value.

Hemolymph Parameters	Adductor Median	Ventricle Median	S	*p*
Cell count (µL)	1070	710	4.0	0.046
Na (mmol L^–1^)	16	16	0.0	1.0
K (mmol L^–1^)	0.53	0.53	1.0	0.317
Cl (mmol L^–1^)	18	18	0.0	1.0
P (mg dL^–1^)	0.85	0.80	0.33	0.564
Ca (mg dL^–1^)	17.50	18.95	4.0	0.046
Mg (mg dL^–1^)	2.75	2.80	2.0	0.157
NH_3_ (µmol L^–1^)	36.3	46.1	1.0	0.317
Protein (mg dL^–1^)	68.2	67.9	0.0	1.0

**Table 2:** The effects of different classes of xenobiotics on the hemocytes and hemolymph of several aquatic invertebrates. The symbol ↑ indicates an increase in value; the symbol ↓ indicates a decrease in value.

Class of Xenobiotics	Species	Ecosystem	LMS ^1^	THC ^1^	Phagocytosis	Biochemical Parameters	Hemocyte Viability and Apoptosis	Refs.
Heavy metals	*Mytilus galloprovincialis*	Marine ecosystem		↓	↓			[26]
	*Orconectes propinquus*	Freshwater ecosystem				↓ Na^+^, Ca^2+^		[27]
	*Portunus pelagicus*	Marine ecosystem				↑ Glucose (after 24 h)		[28]
	*Mytilus edulis*	Marine ecosystem			↑ (10^−9^ to 10^−7^ M)			[29]
	*Tapes philippinarum*	Marine ecosystem	↓		↓			[30]
	*Perna canaliculus*	Marine ecosystem				Sign. altered 25 metabolites		[31]
	*Crassostrea rivularis*	Marine ecosystem		↓	↑			[32]
	*Dreissena polymorpha*	Brackish, freshwater ecosystem			↓		↓ Cell viability	[33]
	*Crassostrea virginica;*	Marine ecosystem					↓ Cell viability; ↑ Apoptosis;	[34]
	*Mytilus edulis*	Marine ecosystem			↓		↓Cell viability	[35]
	*Sinopotamon henanense*	Freshwater ecosystem		↓		↓ Protein content		[36]
	*Palaemon elegans*	Brackish, marine ecosystem		↓				[37]
	*Lamellidens marginalis*	Freshwater ecosystem			↓		↓ Cell viability; ↑ Apoptosis;	[38]
	*Elliptio complanata*	Freshwater ecosystem				↑ Ca^2+^; ↓ Na^+^ No effect on Cl^-^ and K^+^		[39]
Pesticides	*Bellamya bengalensis*	Freshwater ecosystem		↑				[40]
	*Litopenaeus vannamei*	Brackish, marine ecosystem		Sign. difference				[41]
	*Ruditapes philippinarum*	Marine ecosystem		↓				[42]
	*Haliotis tuberculate*	Marine ecosystem	No effect		No effect			[43]
	*Eriocheir sinensis*	Marine, brackish, freshwater ecosystem		↓	↓			[44]
	*Anodonta anatine*; *Lymnea stagnalis*	Freshwater ecosystem	↓					[45,46]
	*Cardisoma armatum*	Terrestrial and marine ecosystem		↓				[47]
	*Biomphalaria glabrata;* *Planobarius corneus*	Freshwater ecosystem	No sign. effect	No sign. effect	↑			[48]
	*Paratelphusa jacquemontii*	Freshwater ecosystem		↓	↓			[49]
	*Mytilus galloprovincialis*	Marine ecosystem				↑ Ca^2+^, NH_3_, Mg^2+^, glucose; ↓ PHOS, urea, Cl^−^, K^+^		[2,5]
Hydrocarbons and oil spills	*Crassostrea gigas*	Marine ecosystem			↓			[50]
	*Crassostrea gigas*	Marine ecosystem		↓	↓			[51]
	*Chlamys farreri*	Marine ecosystem		↓	↓			[52]
	*Haliotis diversicolor*	Marine ecosystem		↓	↓			[53]
Polystyrene microplastics	*Mytilus galloprovincialis*	Marine ecosystem	↓		↓			[54]
	*Mytilus galloprovincialis*; *Mytilus edulis*	Marine ecosystem		↓	↑			[55]
	*Mytilus edulis*	Marine ecosystem			No sign. difference		No sign. difference	[56]
	*Mytilus edulis*	Marine ecosystem	No sign. effect	No sign. effect	No sign. effect			[57]
	*Mytilus galloprovincialis*	Marine ecosystem			↓		↓ Cell viability; ↑ Apoptosis;	[58]
	*Crassostrea gigas*	Marine ecosystem					↑ Hemocytes size	[59]
Amino-modified nanopolystyrene (PS-NH2)	*Mytilus galloprovincialis*	Marine ecosystem		No sign. effect	No sign. effect			[60]
	*Mytilus galloprovincialis*	Marine ecosystem	↓		↓			[61]

^1^ The acronyms “LMS” and “THC” stand for “lysosomal membrane stability” and “total hemocytes count,” respectively.

## Data Availability

Not applicable.
